# Steerable acoustically powered starfish-inspired microrobot†

**DOI:** 10.1039/d3nr03516f

**Published:** 2023-11-10

**Authors:** Cornel Dillinger, Justin Knipper, Nitesh Nama, Daniel Ahmed

**Affiliations:** a Acoustic Robotics and Systems Lab, Institute of Robotics and Intelligent Systems, Department of Mechanical and Process Engineering, ETH Zurich 8092 Zurich Switzerland dahmed@ethz.ch; b Department of Mechanical & Materials Engineering, University of Nebraska-Lincoln Lincoln NE 68588 USA

## Abstract

Soft polymeric microrobots that can be loaded with nanocargoes and driven *via* external field stimuli can provide innovative solutions in various fields, including precise microscale assembly, targeted therapeutics, microsurgery, and the capture and degradation of unwanted wastewater fragments. However, in aquatic environments, it remains challenging to operate with microrobotic devices due to the predominant viscous resistances and the robots’ limited actuation and sensing capabilities attributed to their miniaturization. The miniature size prevents the incorporation of onboard batteries that can provide sufficient power for propulsion and navigation, necessitating a wireless power supply. Current research examines untethered microrobot manipulation using external magnetic, electric, thermodynamic, or acoustic field-guided technologies: all strategies capable of wireless energy transmission towards sensitive and hard-to-reach locations. Nonetheless, developing a manipulation strategy that harnesses simple-to-induce strong propulsive forces in a stable manner over extended periods of time remains a significant endeavor. This study presents the fabrication and manipulation of a microrobot consisting of a magnetized soft polymeric composite material that enables a combination of stable acoustic propulsion through starfish-inspired artificial cilia and magnetic field-guided navigation. The acousto-magnetic manipulation strategy leverages the unique benefits of each applied field in the viscous-dominated microscale, namely precise magnetic orientation and strong acoustic thrust.

## Abstract 2

We introduce a soft acousto-magnetic microrobot leveraging acoustic streaming for propulsion and mixing. The manipulation strategy exploits the distinct advantages of each applied field, precise magnetic orientation, and powerful acoustic propulsion.

## Graphical abstract

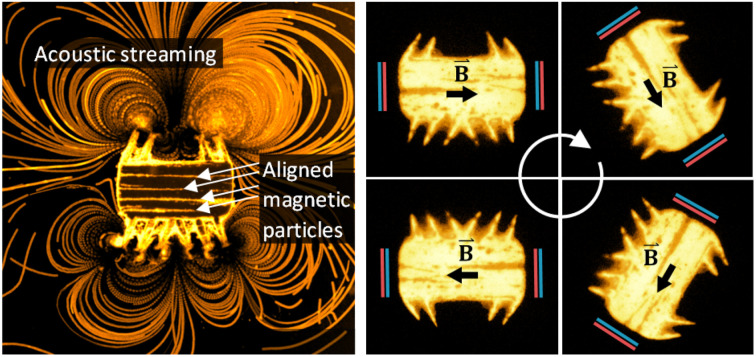

## Introduction

Remote-controlled soft microrobots with wireless capability can be applied in various fields, such as the trapping of microplastics and nuclear waste, cleaning of bacteria-contaminated water, mixing of chemicals, precise assembly of materials, targeted drug delivery, non-invasive microsurgery, and various microfluidic applications.^1–7^ A primary challenge in designing wireless actuation technology in the microscale is the requirement for stable, responsive microrobots characterized by robust propulsion and precise navigational capabilities. At the microscale, aquatic environments are characterized by low Reynolds numbers; inertial effects are negligible, and therefore robotic systems must propel through non-reciprocal motion.^8^ Model bacteria and cells living in such environments, such as *Escherichia coli*, *Helicobacter pylori*, and spermatozoa, have evolved to employ flagellum-based corkscrew-like or whip-like rotational motion for propulsion. Inspired by these microorganisms, numerous methods have been developed for the propulsion of synthetic microscale swimmers, including strategies that utilize external magnetic, electric, and acoustic fields, optical or thermal gradients, and chemical fuels.^9–18^ Each wireless manipulation method leverages its own advantages and is subject to its own limitations while enabling specific tasks in various environments.

Ultrasound-driven actuation principles offer promise due to fluids and tissues having good ultrasound transmission capabilities, are considered clinically safe and biocompatible, are inexpensive to acquire, and can generate large propulsive forces. Large propulsive forces are required to overcome drag forces in highly viscous fluids, to withstand high flow rates, and to achieve high swimming velocities.^19–22^ Based on these capabilities that allow high-precision tasks in hard-to-reach locations such as the human body or within complex machinery, acoustically driven microrobots have generated considerable attention. Particularly microbubble-based acoustic microrobots that can be manipulated to achieve rotational and translational swimming speeds of up to 3.0 mm s^−1^ and 6.0 mm s^−1^, respectively, unveil great potential.^22,23^ These polymeric microrobots asymmetrically encapsulate a microbubble that, when sonicated with ultrasound at its resonant frequency, undergoes periodic expansion and contraction, leading to directional propulsion.^24–26^ However, it remains challenging to steadily control acoustic bubble-based propulsion since the oscillating microbubble's growth rate is subjected to a phenomenon called rectified diffusion.^27^ Over time, the oscillating microbubble grows uncontrollably, and its acoustic responsiveness, *i.e.*, its resonance frequency, changes in an unpredictable manner. Consequently, escaping or collapsing microbubbles cause the acoustic propulsion strategy to be in the long-term temporally unstable. Therefore, research has been conducted towards other acoustic manipulation techniques which do not rely on microbubble actuation. For example, metallic nanorods are demonstrated to perform self-acoustophoresis when exposed to an MHz-range standing wave field due to an emerging acoustic pressure gradient created by a concave-shaped surface on one end.^28^ A major challenge for this type of acoustic actuation is maintaining a standing wave field based on reflections from surrounding interfaces, which, especially in *in vivo* environments, are hard to control. Furthermore, bioinspired flagellated micro/nanorobots exposed to traveling acoustic fields and acoustically induced structural vibrations have demonstrated capabilities to propel objects at the microscale.^29,30^ We recently developed a soft microrobot inspired by starfish larvae that features artificial ciliary bands imitating those observed in the natural model; these bands enable rapid self-propulsion *via* soft structure-acoustofluidic interactions.^31,32^ However, controlled navigation of such systems has not been shown.

In this project, we propose the design of a soft acousto-magnetic microrobot whose manipulation combines precise magnetic orientation with a strong acoustic propulsion strategy. In response to an acoustic stimulus of the environmental fluid, soft polymeric cilia on the microrobot undergo high-frequency small-amplitude oscillations and transduce acoustic energy into propulsive forces. Precise manipulation of the microrobot is facilitated by embedded superparamagnetic particles patterned into parallel lines during the high-throughput fabrication process. The so-induced artificial magnetic easy axis allows for the orientation of the microrobot in an externally applied magnetic field. Due to the unique features enabled by each physical field applied, *i.e.*, precise magnetic orientation and strong acoustic thrust, combined acousto-magnetic manipulation offers great capabilities for advanced remote-control in the microscale. In addition, our microrobots are robust, as they operate reliably in the long term because they don't rely on oscillating bubbles for propulsion. As an additional functionality of these ultrasound microrobots, we showcase viscous mixing through the utilization of its frontal ciliary band.

## Results and discussion

### Bioinspired microrobot design

**Fig. 1:**
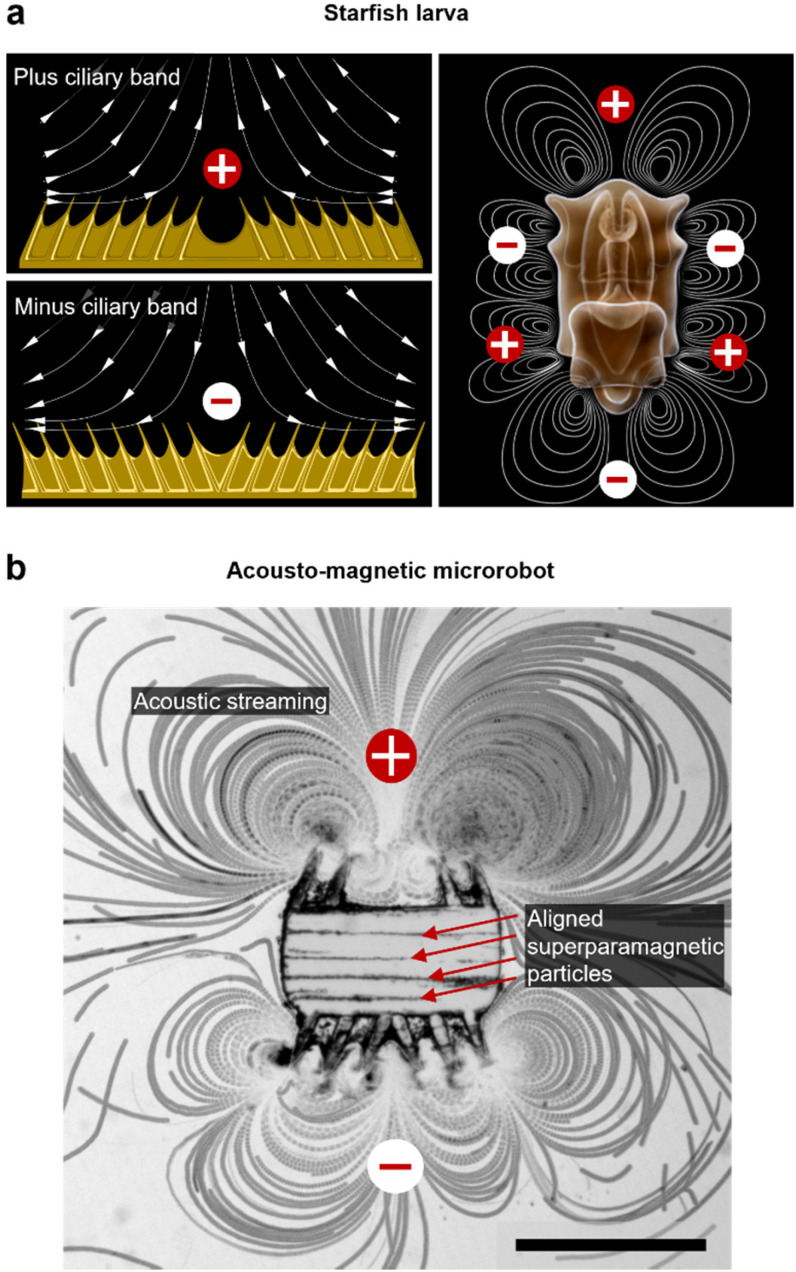
Bioinspired microrobot design. (a) Plus and minus ciliary band configurations covering the body of starfish larvae (left). Excited to oscillate, ciliary bands introduce arrays of vortices in the starfish larva's immediate vicinity (right). (b) A starfish larva-inspired microrobot exposed to an acoustic field with a frequency of *f* = 28.0 kHz and an applied amplitude of 42 volts peak-to-peak (*V*_PP_). Equipped with aligned superparamagnetic particles in the polymeric bulk body, a plus, and minus ciliary band configuration on top and bottom, respectively, the microrobot generates acoustic streaming while being responsive to external magnetic field orientations. The image consists of 400 consecutive video frames captured at a frame rate of fps = 1069 overlaid in a *z*-stack. Scale bar, 250 μm.

The microrobotic design in this project draws inspiration from nature's efficient solutions for microscale propulsion methodologies. Recent studies on starfish larvae have identified them to use two different formations of dynamic self-adjustable ciliary bands, denoted as plus and minus ciliary bands (Fig. 1a). Depending on the condition, a starfish larva employs and oscillates these ciliary bands in formations for swimming or feeding purposes.^32^ According to the arrangement of cilia, a larva establishes a surrounding flow field that either promotes the build-up of few intense vortices that lead to propulsion, or, a maximized number of vortices that favor the entrapment of food in close vicinity (Fig. 1a). Furthermore, the ciliary band formations allow for directional-controlled streaming perpendicular to the larva's body surface. For example, when two ciliary arrays within a band are angled toward each other (a plus band), the fluid is pushed away from the larva's body. In contrast, when two ciliary arrays are angled away from each other (a minus band), the liquid is forced in toward the body. In this study, we built an acousto-magnetic responsive microrobot design based on synthetic imitations of these plus and minus ciliary bands (Fig. 1b).

### Fabrication & experimental setup

**Fig. 2:**
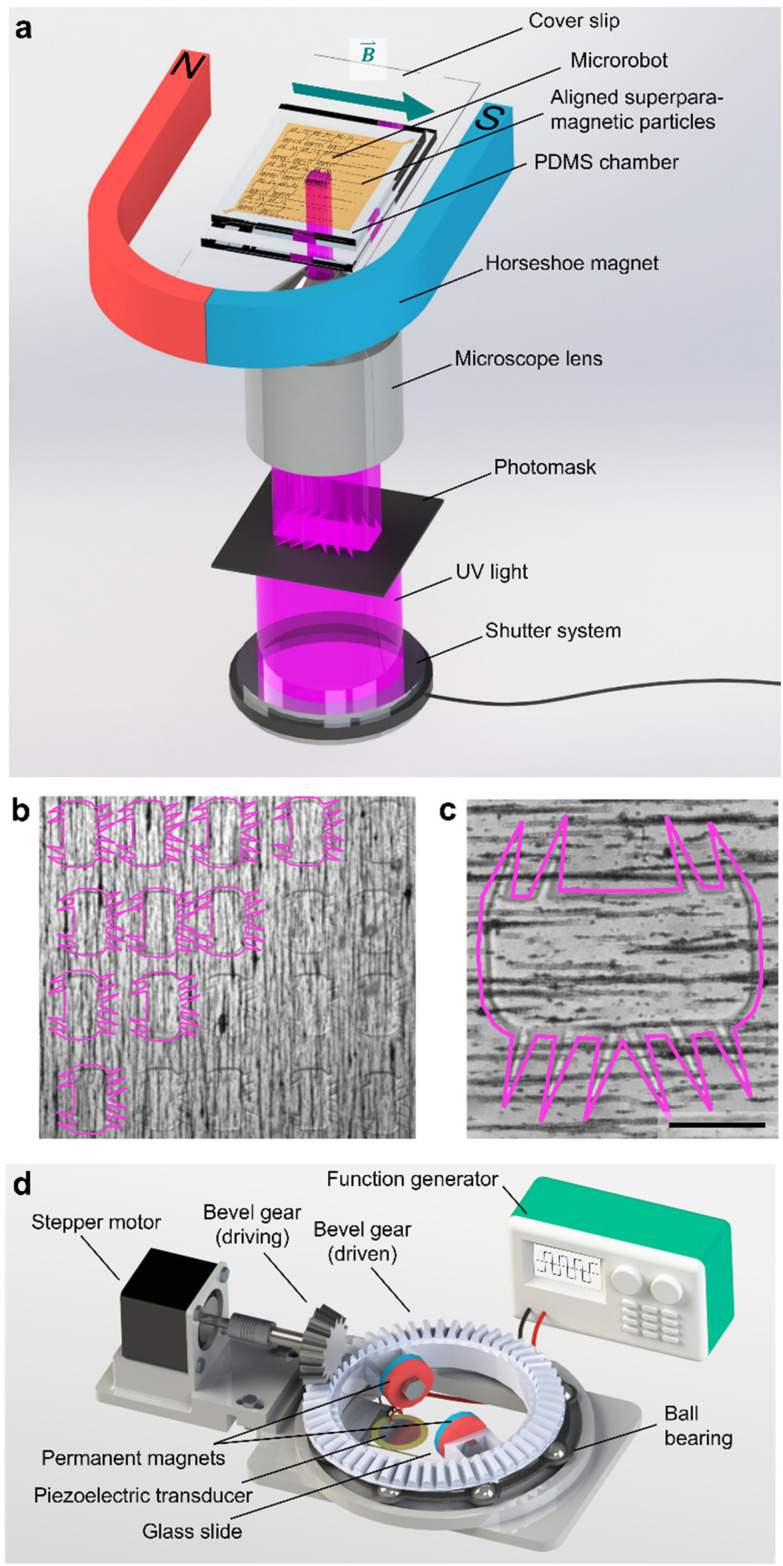
Fabrication and manipulation setup of polymeric acousto-magnetic responsive microrobots. (a) An illustration of the custom-built UV photopolymerization setup installed on an inverted microscope. Superparamagnetic particles align in the PDMS chamber filled with photoresist due to an in-plane placed magnetic field of a horseshoe magnet. (b) and (c) a batch and an individual fully polymerized microrobot still immersed in unpolymerized liquid before the development process. (d) The acousto-magnetic manipulation setup includes a motorized rotational setup of two permanent magnets installed on top of the acoustically stimulated piezoelectric glass slide complex. Scale bar, 100 μm.

We fabricated the polymeric microrobot using an ultraviolet (UV) photopolymerization process installed on an inverted optical microscope (Fig. 2a). A photoresist droplet of 50 μl enriched with 1.5 wt% superparamagnetic microparticles was placed in a molded polydimethylsiloxane (PDMS) fabrication chamber of 33 μm height (see Materials and methods). In this fabrication chamber, suspended superparamagnetic microparticles were patterned into parallel lines due to dipole-dipole interactions induced by an externally applied magnetic field of an in-plane positioned horseshoe magnet. To initiate polymerization, UV light was passed through a high-resolution photomask and focused through the 20× objective of the inverted microscope. An exposure period of 800–1600 ms resulted in the full polymerization of one microrobot. As shown in Fig. 2b, the fabrication method allows for high-throughput production with immediate optical feedback on the quality of the polymerization process, namely the quality of the smallest features, the cilia. A fully polymerized microrobot with superparamagnetic particles aligned along its longest bulk body axis is shown in Fig. 2c. This configuration of the magnetic easy axis is chosen to maximize the magnetic torque an external magnetic field can induce on the microrobot design. Each microrobot consisted of a 285 μm × 135 μm squared bulk body whereon cilia were grown, each with a length of ∼100 μm, a base of 15–20 μm, and a thickness of about 30 μm limited by the PDMS fabrication chamber.

The microrobot was operated on a 25 mm × 75 mm × 1 mm microscope glass slide with a piezoelectric transducer glued to it. Acoustic stimuli were generated by this piezoelectric transducer controlled by a function generator and an interconnected signal amplifier. With the introduction of an artificial magnetic easy axis, the microrobot was forced to align with an external magnetic field that served as a steering guide. The external magnetic field was established by a 3D-printed ball-bearing rotational system carrying two permanent magnets on top of the acoustically activated piezo-glass slide complex (Fig. 2d). The permanent magnets were attached to a circular bevel gear that, in turn, was mounted on a ball bearing. A small driving bevel gear, controlled by an Arduino Uno, enabled this setup to remotely maintain an in-plane rotational magnetic field. The entire setup was mounted on an inverted microscope, and the experimental results were recorded by a CCD or high-speed camera.

### Acoustic propulsion

**Fig. 3:**
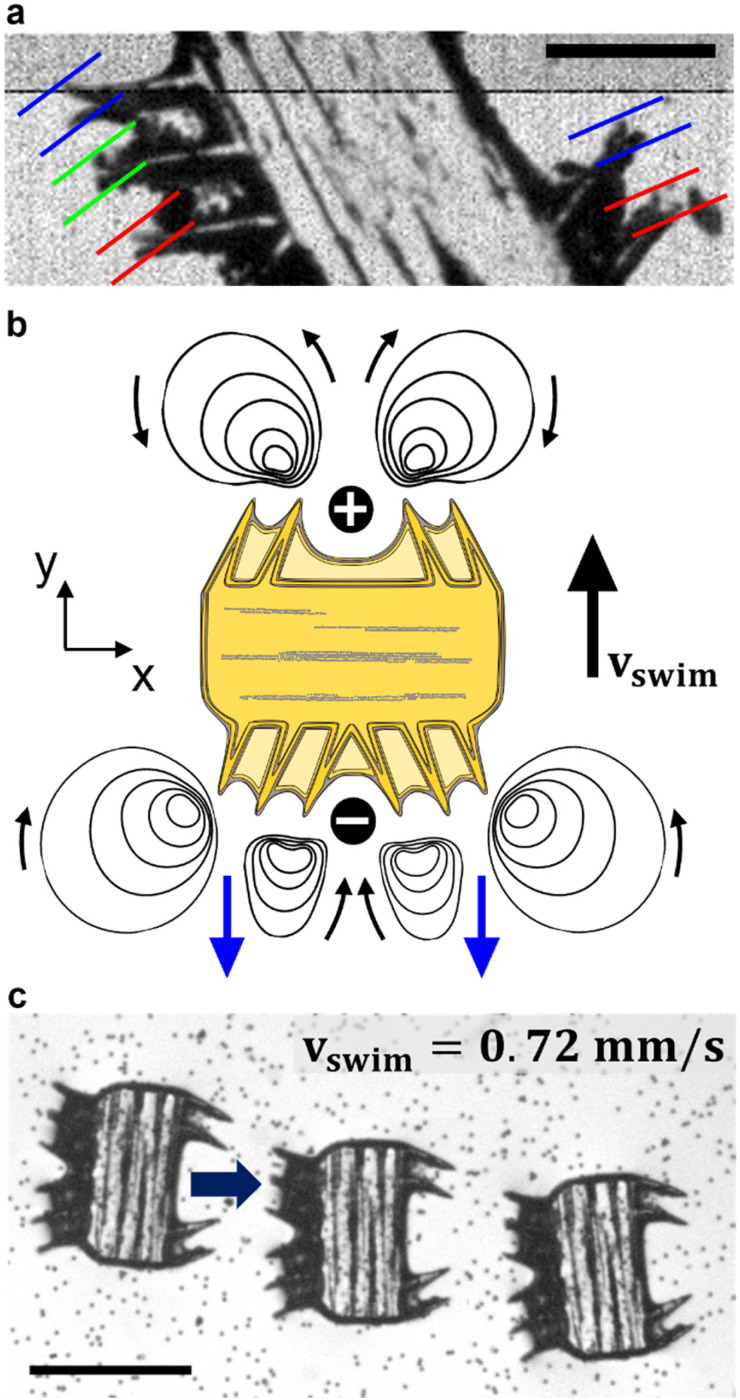
Acoustic response of the microrobot. (a) Cilia oscillation amplitudes on an immobilized microrobot are in the range of *ε* = 7.5–12.5 μm when actuated with a frequency of *f* = 14.1 kHz and an amplitude of *V*_PP_ = 30 V. The image consists of two overlaid video frames captured at a frame rate of fps = 40 413 indicating maximal cilia deflection. (b) Schematic of the acoustic microstreaming with primary propelling streams on the bottom indicated in blue. (c) Left-to-right translational motion of a microrobot acoustically actuated with a frequency *f* = 24.0 kHz and a power amplitude of *V*_PP_ = 30.0 V. Scale bar, 250 μm.

We modulated the excitation frequency of the acoustic field from 5–120 kHz while maintaining the applied voltage at 10–60 volts peak-to-peak (*V*_PP_). When the acoustic stimulation frequency approached the resonant frequency of the piezo-glass slide complex, sufficient acoustic energy was transmitted into the microrobot containing droplet to force the ciliary bands into small-amplitude oscillations (Video S1†). As indicated in Fig. 3a, the ciliary tips’ oscillation amplitudes on a stationary microrobot were determined by overlaying two video frames (captured at a frame rate of 40 413 fps) representing maximum cilia deflection within one oscillation cycle. On cilia actuated with an acoustic amplitude of *V*_PP_ = 30 V and a frequency of *f* = 14.1 kHz, oscillation amplitudes in the range of *ε* = 7.5–12.5 μm were observed. When forced to oscillate, the ciliary bands generated a characteristic vortical steady-state streaming flow called acoustic microstreaming.^33^ This microstreaming is driven by the dissipation of acoustic energy flux in the viscous boundary layer around the microrobot.^34,35^ The boundary layer thickness of the microrobot we report here was calculated as1
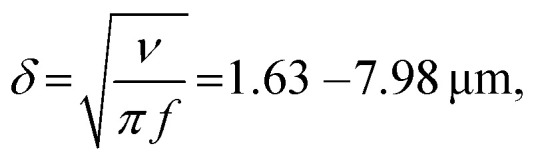
in which we employed *ν* = 10^−6^ m^2^ s^−1^ as the kinematic viscosity of water at room temperature and *f* = 5–120 kHz as the actuation frequency. The counter-rotating vortex flow field directly resulted from viscous dissipations within the boundary layer of the ciliary bands. As shown in the schematic of Fig. 3b, when two ciliary arrays were designed facing each other (a plus ciliary band), the counter-rotating vortices directed the center streamline of the surrounding liquid outwards, analogous to a fluid source. Similarly, when the ciliary arrays were pointed away from each other (a minus band), the center streamline was directed inwards, analogous to a fluid sink. Interestingly, our acoustically actuated cilia oscillate three orders of magnitude faster than their biological counterparts on starfish larvae, but still exhibit qualitatively similar flow patterns and enable similar capabilities.

The Reynolds number calculated for our starfish-inspired microrobot presented in the image sequence of Fig. 3c is found to be Re = *v*_swim_*D*/*ν* ≈ 0.21, where *v*_swim_ = 0.72 mm s^−1^ is the swimming velocity, and *D* = 285 μm is the width of the microrobot. In a low Reynolds number regime as encountered here, the microrobots are located in a viscous-dominated fluidic environment. When viscous effects dominate inertial forces at the microscale, the so-called Scallop theorem for microswimmers states no net motion is achieved by undergoing reciprocal motion only.^8^ Naturally evolved microswimmers, such as *Helicobacter pylori* or *Paramecium*, perform a non-reciprocal type of motion with their corresponding driving element, *i.e.*, corkscrew-like motion of a flagellum or whip-like beating patterns of cilia. Therefore, the here presented polymeric cilia vibrating back and forth at small amplitudes are not meant to induce swimming motion. However, our soft ultrasound ciliary bands are distinct from biological cilia in that they produce flow through reciprocal cilia oscillation. This can be attributed to the high-frequency oscillation of the cilia, which is at least three orders of magnitude higher than what is achieved by their biological counterparts (∼6 Hz).^32^ In such a high-frequency acoustic-actuated system, one must consider the oscillation frequency of the cilia to calculate the corresponding frequency Reynolds number. As demonstrated in previous research, the second-order steady-state system of the governing Navier–Stokes equations in acoustically stimulated systems is driven by time-averaged nonlinear first-order terms that scale with the frequency Reynolds number.^31^ The frequency Reynolds number can be computed as Re_f_ = *2*π*fεL*/*ν* ≈ 471.2–785.4, where *f* = 100 kHz is the excitation frequency, *ε* = 7.5–12.5 μm is the cilia's oscillation amplitude, and *L* = 100 μm refers to a cilia's length. As a result, we encounter a locally inertia-affected system that enables motion at the microscale, even when actuated with reciprocal oscillation patterns.

The interaction between soft ciliary bands and the acoustic waves leads to two phenomena: the development of perturbed (first-order) acoustic fields and streaming fields caused by nonlinear interactions between these perturbed fields. These phenomena result in microrobots experiencing a net propelling force generated by the second-order forcing terms of the Navier–Stokes equations.^31^ Thus, the microrobots propel themselves using oscillating ciliary bands that result in a net propulsion force, arising from the combination of acoustic radiation and streaming forces2
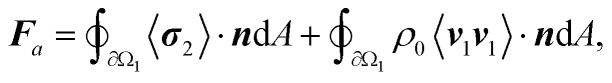
where *Ω*_1_ represents the surface of the microrobot, 〈***σ***_2_〉 is the stress generated by the acoustic streaming, while *ρ*_0_〈***v***_**1**_***v***_**1**_〉 accounts for the nonlinear interactions of the first-order acoustic velocity ***v***_1_ (〈·〉 brackets indicate over one oscillation cycle time-averaged quantities).

However, the calculation of a net propulsive force presents significant challenges. While the widely separated time scales between the first-and second-order fields can be treated through a perturbation approach, a direct numerical simulation of this system remains computationally challenging due to the complex acoustic-fluid-structure interactions that dictate the microrobot motion and the presence of multiple length scales (*e.g.*, distance traversed by swimmer *vs.* the boundary layer thickness) that necessitate extensive meshing efforts. However, using *s* dynamic force balance, where at terminal swimming velocity the acoustic thrust force is balanced by the Stokes drag force (***F***_a_ = ***F***_d_), we calculated the scaling of the force acting on the microrobot as

3 ***F***_d_ = 0.5·*C*_d_·*ρ*·*v*_swim_^2^·*A* ≈ 0.42 nN,

where we assumed our microrobot as a spherical disk (flat plate) with radius *r* = 142.5 μm, drag coefficient *C*_d_ = 12.56, density of water at *ρ* = 998.0 kg m^−3^, swimming velocity *v*_swim_ = 0.72 mm s^−1^, and *A* = 2*·*π*·r*^2^ represents the wetted top and bottom surface area (eqn (S1)†).^36^

### Acousto-magnetic manipulation

**Fig. 4:**
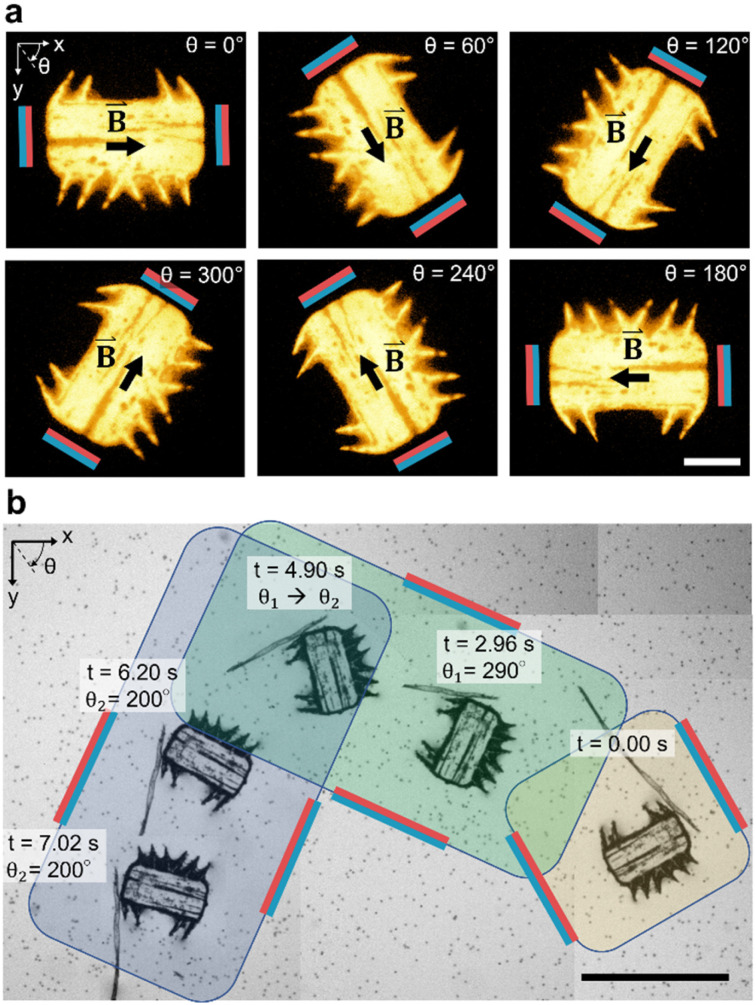
Magnetic manipulation of a soft polymeric microrobot. (a) Controlled rotational motion of a fluorescent microrobot in a glycerol/distilled water mixture (50/50 vol%). Scale bar, 100 μm. In the stitched image sequence (b), the navigation of a microrobot along a 90-degree curved pathway using external magnetic field guidance and acoustic propulsion (*f* = 23.3 kHz, *V*_PP_ = 22.5 V) is shown. Scale bar, 500 μm.

In Fig. 4a, we demonstrate the magnetic orientation of a fluorescent microrobot that aligns with the external magnetic field,

4 ***B***(θ)=*B*_0_·cos(*θ*)·***e***_*x*_ + *B*_0_·sin(θ)·***e***_*y*_

applied by the above-described magnetic setup (Video S2†). The rotational motion is established due to superparamagnetic particles patterned in parallel lines within the microrobot's bulk body (Fig. 1b), which artificially induce a magnetic easy axis into the microrobot. Such a magnetic easy axis in the microrobot's polymeric body, will tend to orientate and align itself with an external magnetic field. We immersed the magnetic-field responsive microrobot in a viscous glycerol/distilled water mixture (50/50 vol%) and measured an average angular speed of *Ω*_water/glycerol_ = 10.6 deg s^−1^ which corresponds to a response time of *t*_water/glycerol_ = 8.5 s for a programmed 90-degree rotation. When immersed in distilled water only, the microrobot's average rotational speed for a 90-degree rotation increases to *Ω*_water_ = 180.0 deg s^−1^ (Fig. S1 and Video S3†). Here, we measured the response time of a stationary microrobot to an external magnetic field-initiated 90-degree rotation to be *t*_water_ = 0.5 s. Subsequently, as illustrated in Fig. 4b, we simultaneously applied acoustic and magnetic fields to manipulate the microrobot along a 90-degree curved pathway (*θ*_1_ = 290°, *θ*_2_ = 200°), guided by a 90-degree counterclockwise rotation of the magnetic field (Video S4†). It is important to note that the microrobot's acoustic response is independent of the relative spatial relationship between the incident acoustic waves and the orientation of the microrobot (Fig. S2†). The microrobots experience a sound field with a wavelength in the order of *λ* = *c*/*f* ≈ 6.4 cm, calculated using the speed of sound in water at room temperature, *c* = 1500 ms^−1^, and an acoustic frequency of *f* = 23.3 kHz. This is a wavelength two orders of magnitude larger than the width of a microrobot (*W* = 285 μm). Consequently, the microrobots experience uniform pressure from all sides.

**Fig. 5:**
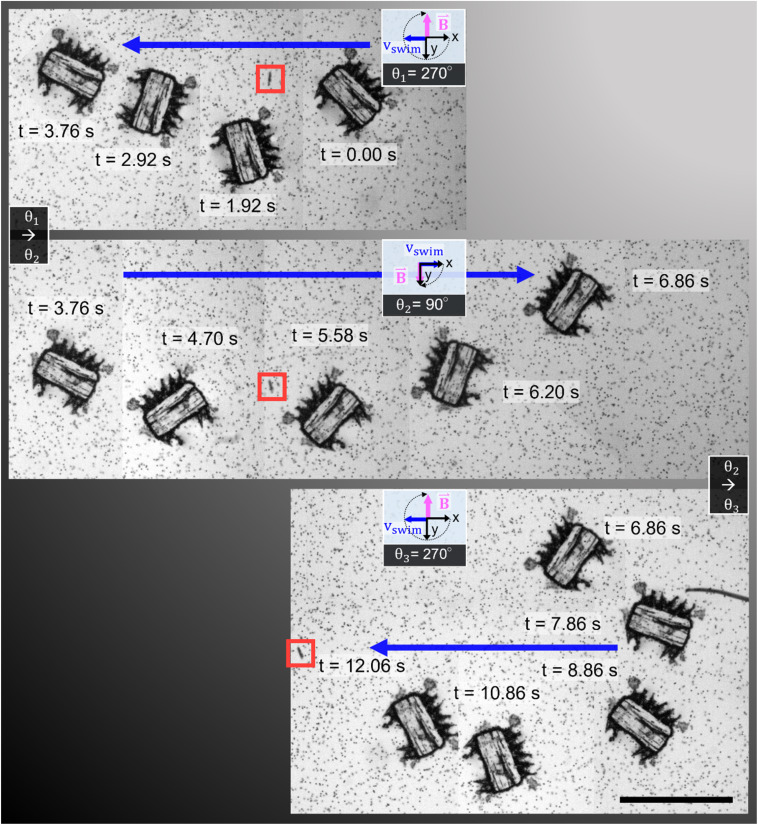
Acousto-magnetic manipulation of a microrobot along multiple turns guided by an external magnetic field and propelled by acoustic thrust (*f* = 23.3 kHz, *V*_PP_ = 30 V). Because of the non-gradient-free magnetic field, drift motion towards the upper image edge during all three horizontal swimming phases was induced. In the middle sequence of the stitched image sequence (red square indicates image reference point), the microrobot translated from left-to-right at an average horizontal speed of *v*_swim_ = 0.75 mm s^−1^ (Video S5†). Scale bar, 500 μm.

In Fig. 5, we demonstrate control of the microrobot through multiple swimming turns guided by consecutively varying magnetic field orientations (*θ*_1_ = 270°, *θ*_2_ = 90°, and *θ*_3_ = 270°) applied during the swimming sequence (Video S5†). In the middle row of the image sequence, the microrobot translates from left to right at an average horizontal velocity of *v*_swim_ = 0.75 mm s^−1^. However, drift motion towards the top edge of the image sequence is visible during all three translations because of the permanent magnets’ non-gradient-free magnetic field. Whenever one of the field-generating permanent magnets was significantly closer to the microrobot, the robot was attracted to it (Fig. S3 and S4†). The force developed by the magnetic gradient can accelerate, decelerate, or induce drift motion to the translational motion depending on the relative position of the microrobot within the external magnetic field (see Fig. S4†). We further evaluated the swimming velocity and found that under acoustic stimulation, the microrobot can accelerate to a swimming speed one order of magnitude higher than achieved when subjected to a magnetic field alone (Fig. S5 and Video S6†). Furthermore, using ultrasonic energy transmission for microrobot propulsion allows the application of high-intensity pulsed ultrasound that can help to overcome critical situations in which a microrobot is stuck to a surface (Fig. S6†). Combining both acoustic and magnetic actuation allows this type of microrobots to be navigated over extended periods of time without relying on delicate acoustic structural resonances of microbubbles or surrounding chamber dimensions.

### Viscous mixing

**Fig. 6:**
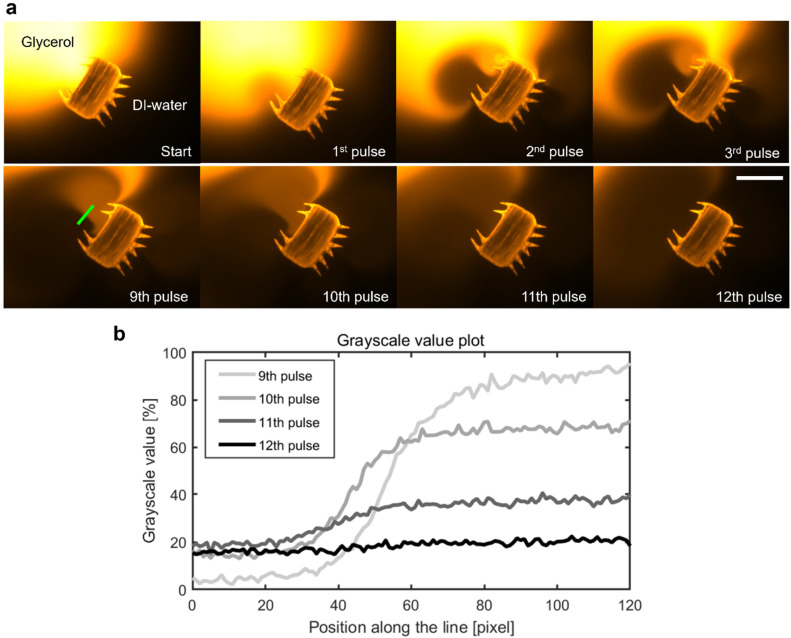
Mixing of viscous glycerol with distilled (DI-) water. (a) A microrobot located at the interface of a fluorescent glycerol droplet (orange) in distilled water (black) is exposed to twelve manually applied pulses of ultrasound with an average pulse duration of *t*_pulse avg_ = 2.61 s, an acoustic frequency *f* = 100.0 kHz, and amplitude *V*_PP_ = 57.5 V (Video S7†). In (b), the mixing process is characterized using normalized grayscale pixel value analysis (min, max intensity) along the green line placed in the main mixing area in front of the microrobot. The two fluid domains are gradually mixed and become a homogenous solution after the 12th pulse of ultrasound, indicated by the uniform grayscale value distribution toward the 12th pulse. Scale bar, 200 μm.

In addition to the precise navigation of the microrobot design, we present a ciliary band's viscous mixing capabilities. A microrobot, penetrating with its plus side ciliary band into the phase of a glycerol droplet immersed in distilled water, is exposed to manually applied continuous square wave pulses of ultrasound (Fig. 6a). Twelve pulses of ultrasound with a frequency *f* = 100.0 kHz, an amplitude of *V*_PP_ = 57.5 V, and an average pulse length of *t*_pulse avg_ = 2.61 s result in the homogenous mixing of the two fluid components in front of the microrobot (see also Video S7†). In the beginning, distilled water is attracted into the glycerol droplet from the left-hand side of the ciliary band and introduces a steep gradient from distilled water to fluorescent-dyed glycerol in front of the microrobot. This gradient remains mostly consistent for the first eight pulses of ultrasound. Afterwards, starting with the 9th pulse of ultrasound, the oscillating plus side cilia arrangement starts to transform this interface between the two fluid domains into a homogenous mixture, visualized in the grayscale value plot of Fig. 6b, representing the pixel values along the green line in Fig. 6a. In the mixing phase, the glycerol is mainly pulled in from the right-hand side of the plus ciliary band whereas the distilled water enters the mixing area from the left-hand side. The mixing process of viscous glycerol (*ν*_glycerol_ = 6.48 m^2^ s^−1^) and distilled water is facilitated by the two counter-rotating vortices generated by the plus ciliary band (see Fig. 1b and 3b). The uniform grayscale value distribution after the 12th pulse of ultrasound sonication suggests homogenous mixing of the two fluid components (Fig. 6b). Mixing at the viscous dominated microscale states a significant challenge due the predominant laminar fluid dynamics that govern the physics in low Reynolds number regimes.^37^ Previous tests have demonstrated that the interface between glycerol and distilled water droplets remains mostly intact for extended periods of time, suggesting minimal diffusion-mediated mixing.^38^ As calculated earlier in the article, the oscillating acoustic ciliary bands locally introduce intermediate frequency Reynold numbers of Re_f_ = *2*π*fεL*/*ν* ≈ 471.2–785.4 that not only enable the propulsion of the microrobot but also viscous mixing capabilities. This result further demonstrates the unique abilities of acoustically stimulated ciliary bands in the microscale and adds a useful functionality to the here presented microrobot design.

## Conclusions

In this work, we demonstrate experimental results that leverage the capabilities of simultaneous acoustic and magnetic manipulation of microrobots and further promote approaches combining the two types of fields. The photolithography-based high-throughput fabrication process of the microrobots is built on an inverted optical microscope and allows for simple magnetization of microscale objects. The acoustic actuation relies on starfish larva-inspired arrangements of soft polymeric cilia that convert acoustic energy flux from fluidic surroundings into propulsive motion based on acoustofluidic interactions. In combination with an externally controlled rotational magnetic field, we manipulated the acousto-magnetic responsive soft microrobot along programmed pathways achieving average swimming speeds up to 0.75 mm s^−1^ using moderate piezo transducer actuation amplitudes of 30 volts peak-to-peak. Additionally, we explored viscous mixing capabilities of an ultrasound-activated ciliary band, thus further illustrating the unique abilities of acoustically stimulated ciliary bands in the microscale. Dually responsive soft composites such as the material utilized here provide an excellent foundation for external field-driven applications of microrobots in sensitive and hard-to-reach aquatic environments. Furthermore, acoustic and magnetic fields are well-established in clinical settings and have been successfully employed for several decades, making acousto-magnetic microrobotics attractive from a physician's perspective. Future work to further advance acousto-magnetic microrobots includes establishing robust 3D manipulation under physiologically relevant flow and fluid conditions in complex microchannel networks. Additionally, increased fabrication resolution may enable a downscaling of the microrobotic design, which can reveal exciting swimming physics since no natural swimmer at the microscale propels itself using such high-frequency oscillation of cilia. From a robotics perspective, the presented microrobot also exhibits interesting characteristics regarding real-time detection using ultrasound imaging. We envision real-time ultrasound imaging of the microrobots using a recently demonstrated method that visualizes induced streaming vortices using the Doppler mode of conventional ultrasonic imaging systems.^39^

## Materials and methods

### Materials

The photoresist utilized to fabricate the microrobot consisted of a 468 mg mixture of 47 vol% PEG-700 (Poly(ethylene glycol) diacrylate with a molecular weight of 700, Sigma-Aldrich), 28 vol% PEG-250 (Poly(ethylene glycol) diacrylate with a molecular weight of 250, Sigma-Aldrich), 14 vol% buffer solution (Tris-EDTA buffer solution, Sigma-Aldrich) and 11 vol% photoinitiator (2-hydrox-2-methylpropiophenone, Sigma-Aldrich). 7.4 mg of superparamagnetic microparticles (Amino paramagnetic particles 1–2 micron, CAT# 18879, Polysciences, Inc.) and 8.5 μL rhodamine solution (Rhodamin B, Sigma-Aldrich) were added to the photoresist mixture to allow for magnetic control and to enable fluorescent imaging of the microrobots. In the viscous mixing experiment, a droplet (5 μL) of glycerol (Sigma-Aldrich) fluorescent dyed with Rhodamin B (Sigma-Aldrich) is used.

### Fabrication and release

50 μL of the photoresist was placed onto a thin (1 mm) PDMS block lying on a 24 mm × 60 mm cover slip (1.5 Spezial, Menzel-Gläser) and was enclosed by another PDMS block containing a molded chamber of 33 μm height. Superparamagnetic microparticles were aligned using an externally applied straight magnetic field (horseshoe magnet). The photoresist was polymerized using a custom-built UV-light polymeriaztion setup. A UV-lamp (Intensilight C-HGFI, NIKON) was connected to an inverted epi-fluorescence microscope (Eclipse Ti, NIKON) and transmitted UV-light through an electric shutter system (VCM-D1, Vincent Associates), a high-resolution photomask (25 400 dpi, Artnet Pro, Inc.) and a 20× objective onto the photoresist. An exposure time of 800–1600 ms was applied using the shutter system, irradiating the negative photoresist, and fully polymerizing the bulk body and the ciliary bands of the microrobot. Upon lifting the top PDMS block, the microrobots can be transferred into an Eppendorf tube by pipetting it with distilled water. Finally, the microrobots were washed and centrifuged three times for 45 s at 6000 rpm to remove unpolymerized photoresist residues.

### Magnetic torque generation

A motorized rotational system to control the position of two permanent magnets was 3D-printed (Creality Ender-3 Pro) after being designed in Solidworks. Using screws, the magnets were attached to a bevel gear, which in turn was mounted on a ball bearing apparatus, allowing it to rotate in plane of the microscope stage. The magnet-carrying bevel gear was driven by a smaller bevel gear mounted on the drive shaft of a stepper motor controlled by an Arduino Uno.

### Acoustic setup

A piezoelectric transducer (Piezoelectric Diaphragm 27 mm leaded, Murata) was glued to a 25 mm × 75 mm × 1 mm microscope glass slide (Menzel-Gläser) using a two-component glue (2-K-Epoxidkleber, UHU Schnellfest). The piezoelectric transducer was connected to an electronic function generator (AFG3011C, Tektronix) which in turn was connected to an amplifier (HF-Amplifier, Digitum-Elektronik). The acoustic setup was then mounted in the 3D printed magnetic manipulation system and placed on an inverted microscope (Eclipse Ti, Nikon or Axiovert 200M, Zeiss). Results of the experiments were captured using a high-speed camera (Chronos 1.4, Kron Technologies) and/or a fluorescent camera (Coolsnap EZ, Photometrics).

## Author contributions

D. Ahmed and C. Dillinger conceived the project idea. C. Dillinger and J. Knipper were responsible for the experimental work. C. Dillinger was leading the article writing process. D. Ahmed and N. Nama are advisors who provided theoretical support and article writing suggestions.

## Conflicts of interest

There are no conflicts to declare.

## Supplementary Material

NR-016-D3NR03516F-s001

NR-016-D3NR03516F-s002

NR-016-D3NR03516F-s003

NR-016-D3NR03516F-s004

NR-016-D3NR03516F-s005

NR-016-D3NR03516F-s006

NR-016-D3NR03516F-s007

NR-016-D3NR03516F-s008
