# Supplementary material for: Steerable acoustically powered starfish-inspired microrobot
Source: Nanoscale. 2023 Nov 10;16(3):1125–34. doi: 10.1039/d3nr03516f (PMC10795801; doi:10.1039/d3nr03516f)
Supplement: NR-016-D3NR03516F-s001 [file NR-016-D3NR03516F-s001.pdf]

This journal is © The Royal Society of Chemistry 2023

Supplementary Information

Steerable Acoustically Powered Starfish-inspired Microrobot

Cornel Dillinger,[a] Justin Knipper,[a] Nitesh Nama[b] and Daniel Ahmed[a]

Supplementary Equation:

Drag coefficient (C_d) calculation according to Tomotika and Aoi[1]:

$$C_d = \frac{4\pi}{RS}\left[1 - \frac{1}{S}\left(S^2 - S - \frac{5}{12}\right)\frac{R^2}{128} - \frac{1}{S^2}\left(S^4 + \frac{1}{12}S^3 - \frac{23}{24}S^2 - \frac{133}{360}S - \frac{25}{144}\right)\frac{R^4}{128^2}\right] \approx 12.56 \qquad \text{(S1)}$$

with R = $v_{swim}D/\nu$ ≈ 0.21 and S = 3.1954 – ln(R) ≈ 4.76, where v_{swim} = 0.72 mm s^{-1} refers to swimming, D = 285 µm is the characteristic length scale corresponding to the microrobot's bulk body width, and ν = 10−6 m^2 s^{-1} is the kinematic viscosity of water at room temperature.

Reference:

[1] S. Tomotika, T. Aoi, *Q. J. Mech. Appl. Math.* **1953**, *6*, 290.

Supplementary Figures:

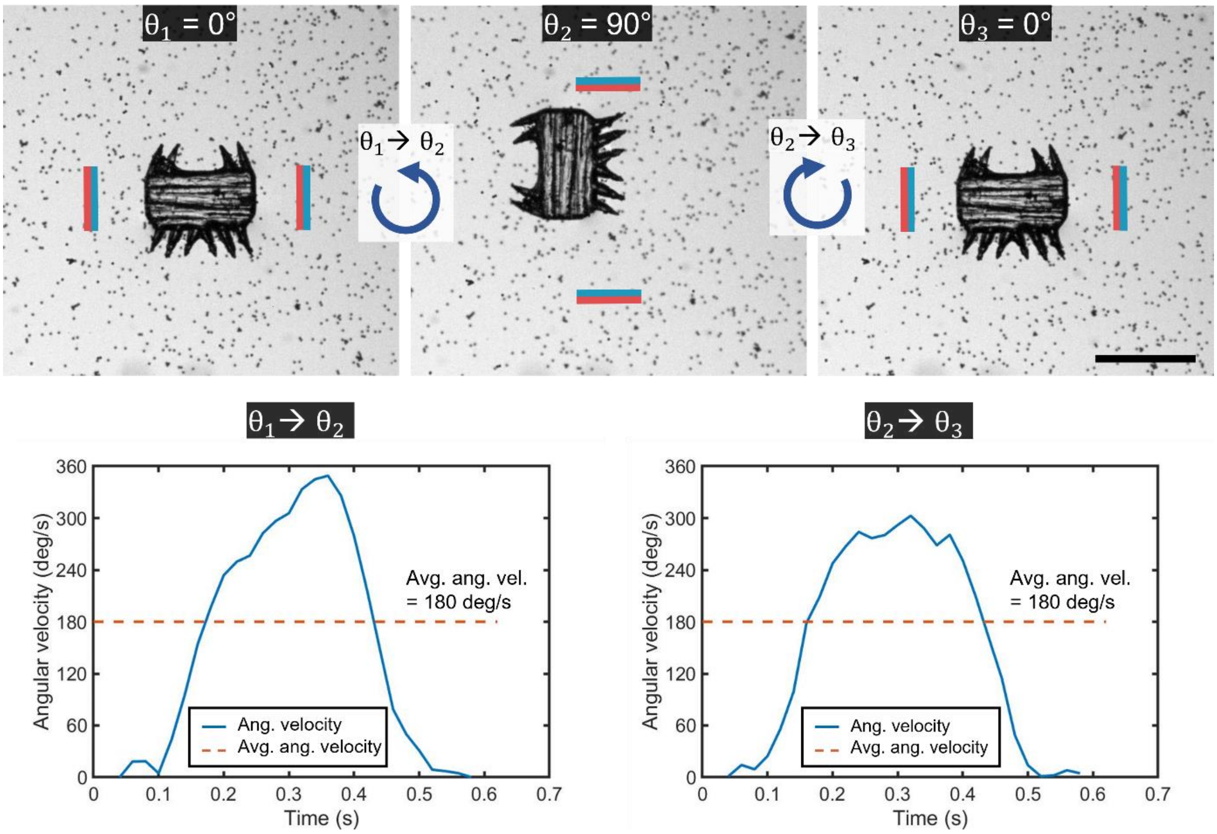

Fig. S1 Microrobot orientation in an external magnetic field. A microrobot is manipulated to undergo two consecutive 90-degree turns, first in clockwise, then in counterclockwise rotational direction (Video S3). Immersed in distilled water, the microrobot fulfills a 90-degree rotation in response to the external applied magnetic field in ~ 0.5 s and rotates with an average angular velocity of Ω_{water} = 180.0 deg s^{-1}. The rotational motion was analyzed using image analysis tools of the ImageJ software. Scale bar, 250 µm.

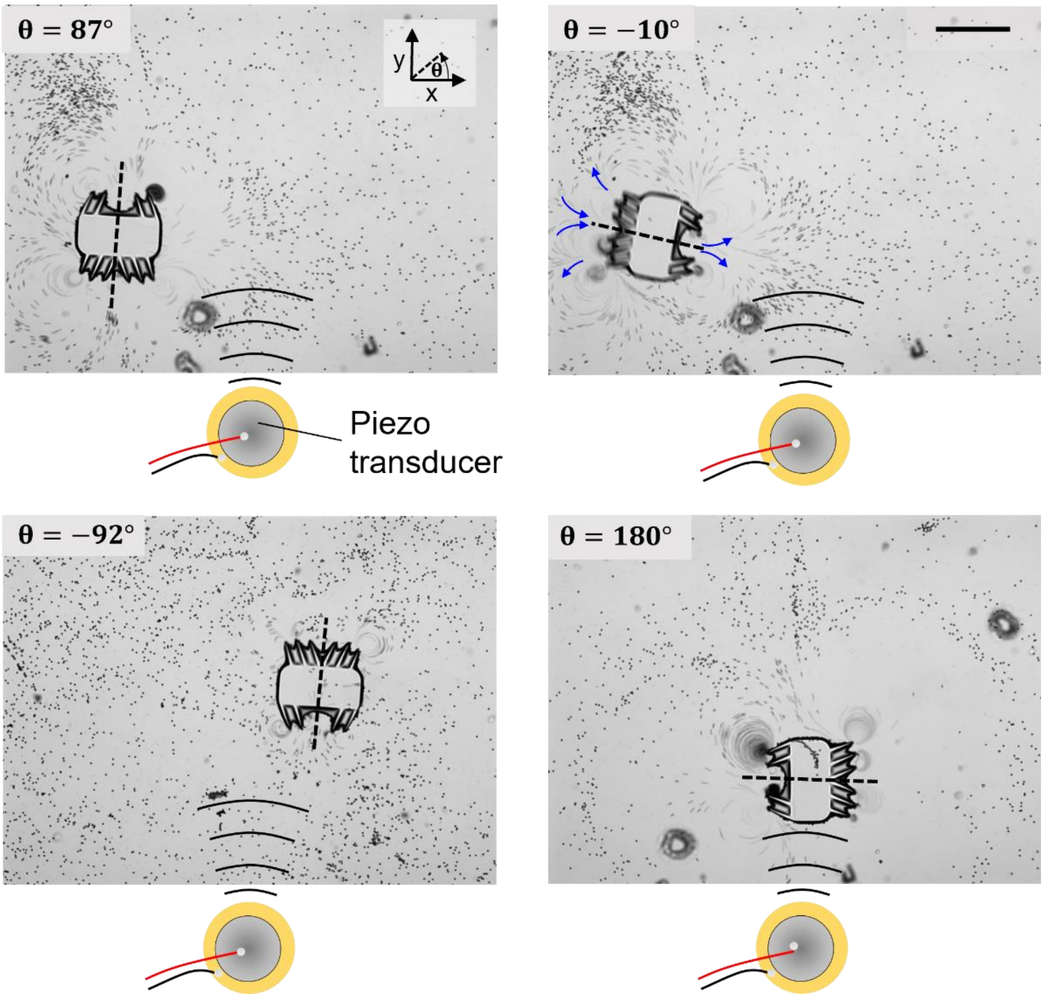

Fig. S2. Orientation-independent acoustic response of the acoustic microrobot stimulated by a piezoelectric transducer located at the bottom image edge. A microrobot in four different orientations with respect to the same acoustic source exhibits the same acoustic response in form of acoustic streaming. Scale bar, 250 µm.

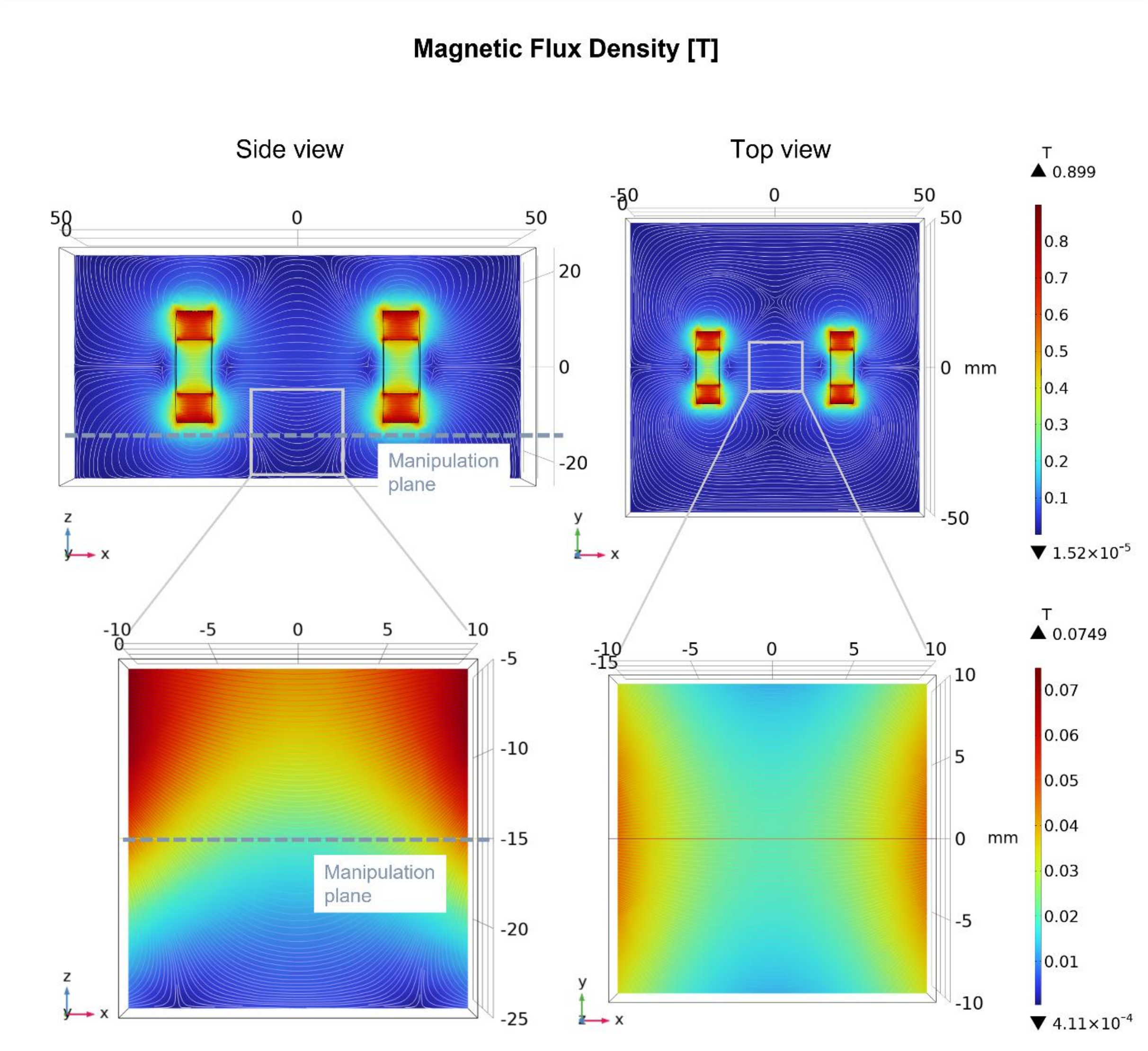

Fig. S3 Magnetic field simulation (COMSOL 6.1). A numerical simulation of the magnetic field generated by the two opposing ring-shaped permanent magnets shows a non-gradient free magnetic field intensity. The insets, indicated with a grey rectangle (left) and a grey square (right), illustrate the relatively low magnetic field gradient in the microrobot manipulation area compared to the strong permanent magnets. In the COMSOL Multiphysics software, the "Magnetic Fields, No Currents" module and the "Remanent flux density" magnetization model were used. The material of the permanent magnets is set to N35 (Sintered NdFeB) and the model mimics all geometrical parameters of the experimental setup embedded in an environmental air domain.

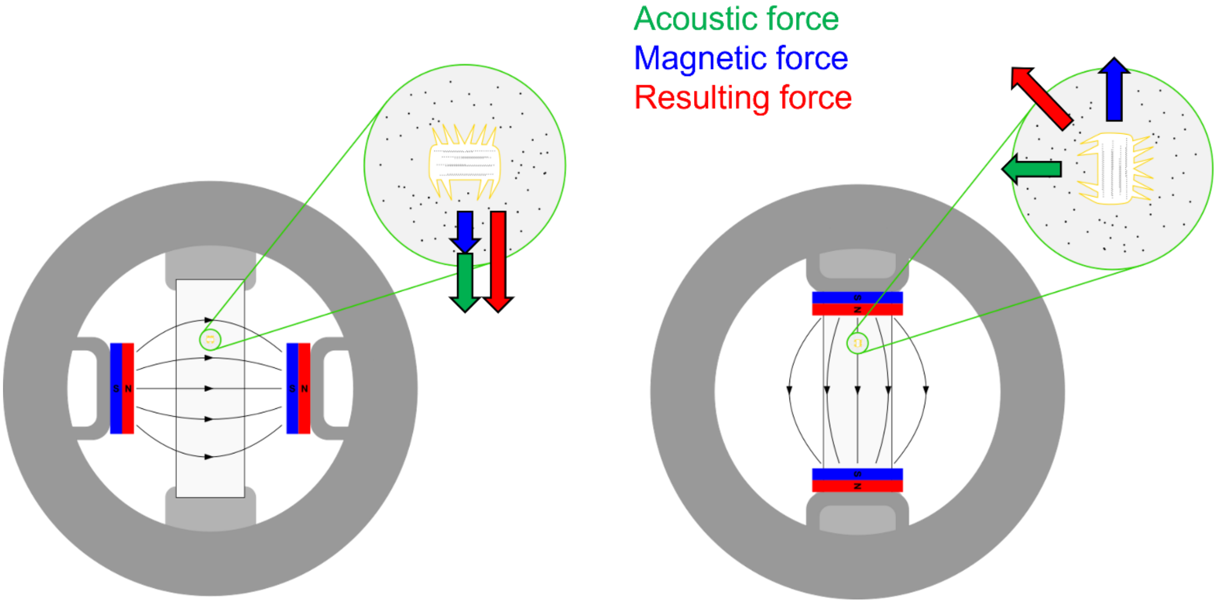

Fig. S4 Magnetic field gradient-induced drift motion. The rotating magnetic field setup is built using two permanent ring-shaped magnets, generating a non-gradient free magnetic field (Fig. S2). Depending on the location, the robot under manipulation is exposed to an acoustic thrust force as well as a magnetic gradient conditioned force that results in weak drift motion (Video S5).

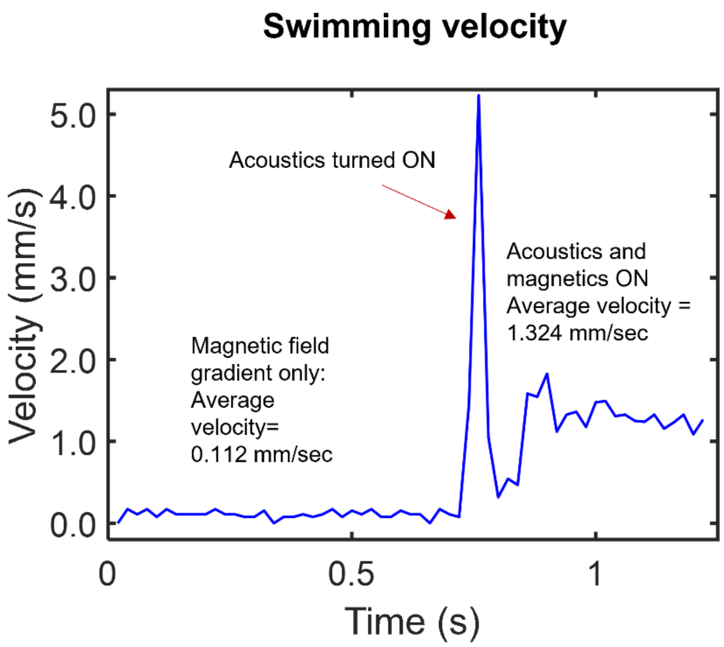

Fig. S5 Acoustic vs. magnetic field gradient induced swimming velocity. Without acoustic stimulation, a microrobot experiences a magnetic field gradient induced force that leads to a terminal average swimming velocity of ~ 0.112 mm s^{-1}. When acoustics is turned on, the microrobot experiences two external field-induced forces, i.e., magnetic, and acoustic actuation (frequency f = 23.3 kHz, amplitude of V_{PP} = 30 V) act combined on the microrobot and can lead to a terminal average swimming velocity of ~ 1.324 mm s^{-1} (Video S6). That is approximately one order of magnitude higher and corresponds to ~ 4.4 body lengths s^{-1} (body length ≈ 300 µm includes bulk body and protuberant cilia on each side). The velocity analysis was performed using the Manual Tracking plugin of the open-source software platform ImageJ.

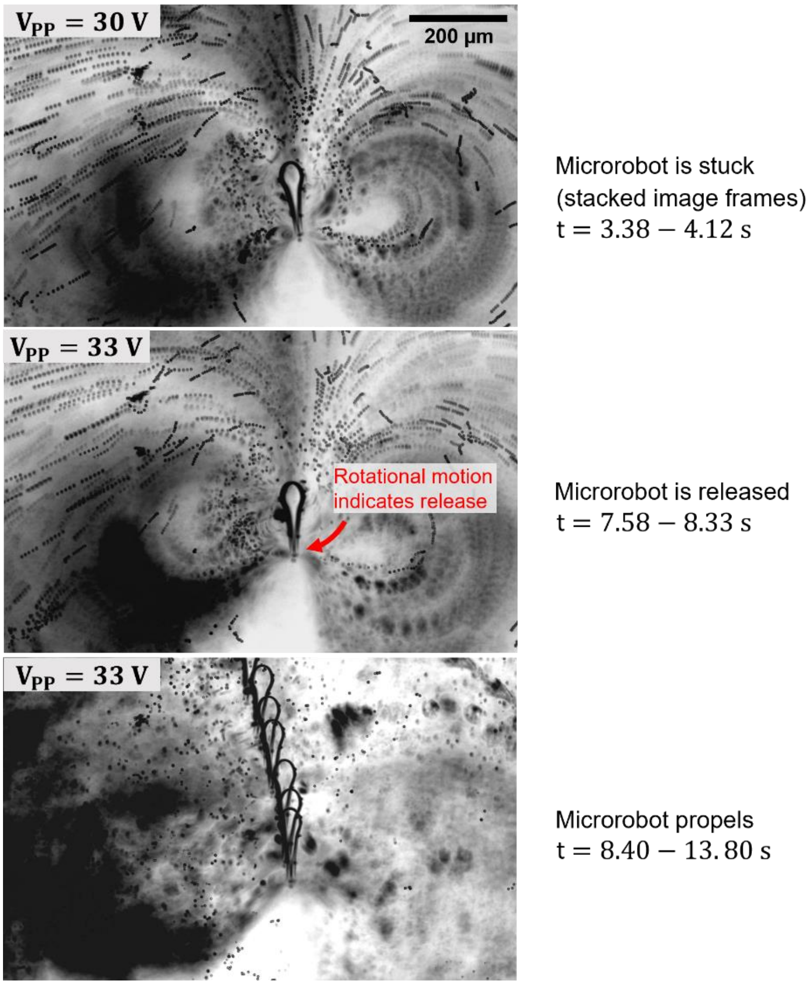

Fig. S6 A polymeric microrobot is released from the ground upon the application of an increased acoustic power amplitude of $V_{pp} = 33.0$ V. Scale bar, 200 µm.

Supplementary Videos:

Video S1: In Video S1, the cilia oscillation amplitude of an immobilized microrobot is captured. The recording frame rate was *fps* = 40413, and the video is played at frame rate of fps = 4. The microrobot was actuated using an acoustic field with frequency *f* = 14.1 kHz and an amplitude of V_{PP} = 30 V.

Video S2: In Video S2, we present a microrobot's response to a continuously rotating external magnetic field. The microrobot is immersed in a viscous glycerol/distilled water mixture (50/50 vol.-%).

Video S3: In Video S3, we present a microrobot's response to two consecutive 90-degree induced turns of the externally applied magnetic field. The microrobot is immersed in distilled water and completes the programmed rotation within ~ 0.5 s.

Video S4: In Video S4, we demonstrate a swimming 90-degree turn under magnetic and acoustic stimulation. The microrobot is acoustically actuated with a frequency *f* = 23.3 kHz and an amplitude of V_{PP} = 22.5 V.

Video S5: In Video S5, a microrobot is acousto-mangtically manipulated along multiple turns to show precise maneuverability. The established acoustic field consists of a frequency *f* = 23.3 kHz and an amplitude of V_{PP} = 30 V.

Video S6: Using Video S6, a swimming speed comparison between magnetic and acousto-magnetic actuation is done. At first, the microrobot is exposed to the magnetic field gradient-induced force only. Then, acoustics is turned on at a frequency of *f* = 23.3 kHz and an amplitude of V_{PP} = 30 V.

Video S7: In Video S7, a microrobot located at the interface of a fluorescent glycerol droplet in distilled water is exposed to twelve manually applied pulses of ultrasound with an average pulse duration of $\mathrm{t_{pulse\ avg}}$ = 2.61 s, an acoustic frequency f = 100.0 kHz, and amplitude $\mathrm{V_{PP}}$ = 57.5 V. The two fluid domains are gradually mixed and become a homogenous solution.
